# The Burden of Prudery

**Published:** 1911-03

**Authors:** G. Henri Bogart

**Affiliations:** Brookville, Ind.


For Texas Medical Journal.

### The Burden of Prudery.

BY DR. G. HENRI BOGART, BROOKVILLE, IND.

One of the terrible tragedies of society has just passed across my view; one of those unsuspected, all too common elements of life; many fold more tragic than death.

Yes, it is a woman. She is one of the noblest women I have ever met; intellectual to high degree—pure, strong, progressive.

She possesses the remnants of a glorious vitality, else she had not lived to inspire this paper. I learn that, as a housewife, she is a model; she reads and digests the foremost thought of the day, and, with it all, finds time to assist her husband in his business, not from mere acquisitiveness, but to "be up and doing."

She has been married fourteen years, and her desire for maternity is such as could be expected from the character which I have so feebly tried to portray. And the world needs progeny from such as she, with her natural mental and physical equipment to bring a splendid heredity, to which her capableness would have been a guiding star—and she will never be a mother.

She wrote me for a consultation, as to the dictum of her physicians concerning her sterility, and I had to confirm the verdict, and to go further and enjoin that she should safeguard her own strength, lest she succumb. In a voice vibrant with tears, she ejaculated, forgetting my presence, "Oh, my mother, my mother, why in God's name did you lay this burden upon me?"

The sight of a soul, torn, and laid bare by the sentence of hopelessness is trying, but there was nothing for me to do but to sit powerless and allow the paroxysm to pass.

She told me of the home-life of her girlhood—aristocratic, secluded from the rough blasts and contacts of the world, with the safeguards thrown out to prevent straying.

In her fifteenth year she first menstruated, and when she arose that first morning, and discovered the flow, she was horrified at the unknown thing that had come to pass. The mother discovered the conditions, and led the skrinking girl into the chamber, scolded her for soiling the bed linen, gave her napkins, and told her how to apply them, and nothing more. That period was a nightmare of terror and humiliation to the sensitive child, and succeeding flows were but little better.

She had noted that a neighbor who had suffered a hemorrhage had been packed into ice, to restrain the flow of blood, and so, in winter, she would sit on snow banks, and in summer use cold packs or the sitz baths, and this continued for two years. Then a comrade explained the phenomenon to her, but it was too late. But for her wonderful vitality, she had needed an undertaker, but, as it is, she is an invalid, and her life is ruined. She is not a whining hypochondriac, still she breaks down, once or more times annually, and the family knows of it when she is found collapsed.

As an instance of her innate mother love, one who knows her well told me this anecdote. One day she was standing on the wooden platform of a country railroad station while a freight was pulling up to the siding to allow a train to come in. A

lout of a boy tried to jump onto the side ladder, and fell into the narrow space between the platform and the wheels, one leg being cut off as he fell. He began struggling to rise, when she threw herself onto the planking, reached down and held him until the train had passed, else he had been cut in pieces. Her own hat was torn away, so close was the space; then her beautiful gown, bloody and grimy, she fainted. On being restored, she was asked what made her do it, and she answered, "I thought so of his mother." She had never before seen the boy.

And this woman so grandly equipped by nature for motherhood, and so welling over with mother love, shall not leave to the world the heritage of a line of descendants, because the damning shackles of prudery held that "ignorance is innocence."

I am proud of the race every time I open one of the modern magazines and find therein those truths of eugenics, the true rendition of "Know thyself," for it shows that the real world of man and woman is coming into the reality of life.

The double sexual standard and the guarded teaching of ignorance have strewn the walks of earth with countless wrecks, which our profession, from the summits of attainment, may never restore.

Doctor, I would that I could take this story, with all of heartache that it means to me, and carry its fullest import into the soul depths of every mother on earth, but I can not.

I can beg you, each and sundry, however, to use the social and directive power, which the prestige of your profession gives to you, that you join in teaching the mothers what they should teach their daughters.

The world movement, which is seeking to safeguard our race by the plain truths of sexuality, is a new revelation. You are its ministers, and let us every man work for uplift.

